# Multi-feature data repository development and analytics for image cosegmentation in high-throughput plant phenotyping

**DOI:** 10.1371/journal.pone.0257001

**Published:** 2021-09-02

**Authors:** Rubi Quiñones, Francisco Munoz-Arriola, Sruti Das Choudhury, Ashok Samal

**Affiliations:** 1 Department of Computer Science and Engineering, University of Nebraska-Lincoln, Lincoln, Nebraska, United States of America; 2 School of Natural Resources, University of Nebraska-Lincoln, Lincoln, Nebraska, United States of America; 3 Department of Biological Systems Engineering, University of Nebraska-Lincoln, Lincoln, Nebraska, United States of America; COMSATS University Islamabad, Wah Campus, PAKISTAN

## Abstract

Cosegmentation is a newly emerging computer vision technique used to segment an object from the background by processing multiple images at the same time. Traditional plant phenotyping analysis uses thresholding segmentation methods which result in high segmentation accuracy. Although there are proposed machine learning and deep learning algorithms for plant segmentation, predictions rely on the specific features being present in the training set. The need for a multi-featured dataset and analytics for cosegmentation becomes critical to better understand and predict plants’ responses to the environment. High-throughput phenotyping produces an abundance of data that can be leveraged to improve segmentation accuracy and plant phenotyping. This paper introduces four datasets consisting of two plant species, Buckwheat and Sunflower, each split into control and drought conditions. Each dataset has three modalities (Fluorescence, Infrared, and Visible) with 7 to 14 temporal images that are collected in a high-throughput facility at the University of Nebraska-Lincoln. The four datasets (which will be collected under the *CosegPP* data repository in this paper) are evaluated using three cosegmentation algorithms: Markov random fields-based, Clustering-based, and Deep learning-based cosegmentation, and one commonly used segmentation approach in plant phenotyping. The integration of *CosegPP* with advanced cosegmentation methods will be the latest benchmark in comparing segmentation accuracy and finding areas of improvement for cosegmentation methodology.

## Introduction

To ensure that crop production will sufficiently satisfy the needs of a human population that is expected to grow to more than 9 billion by 2050 is a tremendous challenge for plant science and agriculture [1]. This goal is challenging primarily because the average rate of crop production is increasing only 1.3% per year, and it cannot keep pace with population growth. Therefore, it is important to achieve efficient, automatic [2, 3], and reliable physical and cyber infrastructures to enable self-managing and sustainable farming [4]. Researchers will need to identify a plants’ ability to respond and adapt to environmental changes. Fahlgren et al. [5] states that the recent developments in high-throughput phenotyping can be leveraged to aid in the maintenance and improvement of crop yield. Previously, researchers used manual interventions to measure plant phenotypes causing a disruption to the plant growth. Therefore, it is imperative that data collection of plants is fast, efficient, and accurate. Collecting data via a high-throughput imaging system will yield more complex datasets versus the current method of manual data collection [5]. With the potential of creating complex, object-evolving datasets that can synthesize the time cycle of a plant, using high performing algorithms is crucial.

Segmenting an object from a background is considered a low-level (initial/beginning) image processing method that uses thresholding [6]. There are works that currently use machine learning and deep learning algorithms to acquire training data in plant phenotyping [7]. However, the training obtained is specifically meant for datasets with limited features. Rother [8] argued that complete automatic segmentation is possible but prone to error, and interactive input or fusion with other modalities, is normally needed to correct those errors. Consequently, it is a method that is dependent on the amount of training data available in a given dataset and the process to obtain training data is tedious, long, and manually done by humans.

Existing cosegmentation algorithms have been classified by Merdassi [6] into eight different categories: Markov random fields-based cosegmentation (MRF-Coseg), Co-saliency-based cosegmentation, Image decomposition-based cosegmentation, Random Walker-based cosegmentation, Maps-based cosegmentation, Active contours-based cosegmentation, Clustering-based cosegmentation (Cl-Coseg) and Deep learning-based cosegmentation (DL-Coseg).

Below is a list for the hypothesized impact of our datasets on the performance of three chosen algorithms based on the code availability and ability to handle large collections of images.

1. **MRF-Coseg** [9]: for its use of inter-group information passing.
  *Hypothesis*: Will benefit temporal analysis.
  *GitHub Code*: MIG.
  *Commit Used*: 001093 on April 20, 2017.
2. **Cl-Coseg** [10]: for its use of clustering using overlapping information.
  *Hypothesis*: Will benefit temporal and multi-perspective analysis.
  *GitHub Code*: Subdiscover.
  *Commit Used*: f01e63f on December 24, 2014.
3. **DL-Coseg** [11]: for its use of self-supervised learning.
  *Hypothesis*: Will benefit multi-modality analysis.
  *GitHub Code*: DeepCO^3^.
  *Commit Used*: 7c14b18 on April 29, 2019.

This paper will introduce 1) a benchmark analysis [12], 2) the Cosegmentation for Plant Phenotyping, *CosegPP*, data repository, and 3) a comprehensive study to establish a benchmark between the integration of plant phenotyping and cosegmentation. *CosegPP*’s objects, i.e., the plants, vary in color and texture as they grow to maturity. Due to its growth in size, the plants also vary the background due to the camera’s zoom ability to capture the full plant. Therefore, we hypothesize that cosegmentation will have greater success with mature plants that have more surface area in the visible light modality.

By implementing cosegmentation in the plant phenotyping field, we make the following contributions:

1. First, we introduce a benchmark analysis establishing the performance and gaps in computer science of current cosegmentation algorithms and datasets.
2. Second, we construct *CosegPP*, a data repository consisting of four datasets. *CosegPP* has technical features including temporal, multi-perspective, and multi-modal, and plant features such as drought vs control conditions, and species type. In total there are 500 images and 48 groups. Each image has a ground truth image of the segmented object.
3. Third, we present a comprehensive study that introduces the first coupling between cosegmentation algorithms and a plant phenotyping dataset (*CosegPP*).

## Related work

### Cosegmentation datasets

There have been a few datasets proposed in the field of cosegmentation [11, 13–17] as shown in Table 1. The Microsoft Research Cambridge (MSRC) dataset was one of the first datasets to be created for recognizing objects in a group of images. PASCAL Visual Object Classes (PASCAL-VOC) and Interactive Cosegmentation (iCoseg) followed five years later with much larger datasets increasing their number of groups and total count of images. PASCAL-VOC had 20 groups with 10,103 images (approximately 505 images per group) and iCoseg had 38 groups with 643 images (approximately 17 images per group). During the next few years, the Internet and Flicker Media Forensic Challenge (FlickerMFC) dataset was released, in which the images were collected online to obtain large training datasets with pixel-level masks. This significantly increased the number of images and range of foreground objects per image. These datasets characteristics are shown in Table 1.

**Table 1:** Characteristics of existing cosegmentation datasets with our four proposed datasets. For our analysis, we will use iCoseg, MSRC, and Internet.

Dataset	Year	#Groups	#Images	#Foreground Objects	Object Type
*iCoseg* [14]	2010	38	643	1-3	Landmarks, sports, animals, misc.
*MSRC* [13]	2005	8	240	1-8	Animals, foliage, man-made structures, misc.
*Internet* [15]	2013	3	18,618	1-12	Airplane, car, horse
*FlickrMFC* [16]	2012	14	263	3-8	Animals, people, foliage, man-made structures
*PASCAL-VOC* [17]	2010	20	10,103	1-5	People, animals, vehicles, indoor objects
***Buckwheat-C-1 (ours)***	2021	12	84	1	Plants
***Buckwheat-D-1 (ours)***	2021	12	84	1	Plants
***Sunflower-C-1 (ours)***	2021	12	168	1	Plants
***Sunflower-D-1 (ours)***	2021	12	168	1	Plants

Although these datasets have advanced cosegmentation methodology, they lack in object temporal characteristics. *CosegPP* provides a temporal aspect of the object (plant) which creates more unique size, color, and texture features for a single object. This is a challenge for cosegmentation since previous analyses have been focused on segmenting a wide range of large objects. More so, objects (such as people, and animals) are different sizes, and colors, and previous datasets have focused on solely children or adults as a group.

### Cosegmentation methods

Current cosegmentation methods have achieved promising performance when attempting to segment foregrounds from an image. This paper investigates three methods from the DL-Coseg, Cl-Coseg, and MSF-Coseg category. The latest DL-Coseg method is Deep Instance Co-Segmentation by Co-Peak Search and Co-Saliency Detection (DeepCO^3^) [11] where it attempts to segment all the foreground objects per image using instance cosegmentation. This method uses a Convolutional Neural Network (CNN)-based network in its instance mask segmentation sub task while using four datasets: MS COCO [18] (divided into two datasets due to its size), PASCAL VOC [17, 19], and SOC [20]. The authors used CorLoc^r^ and mean average precision (mAP) to evaluate their results. Chen’s Subcategory Discovery (Subdiscover) method [10] is a Cl-Coseg method that uses the Internet database [15] to automatically discover objects and their segmentations from noisy images. Their metrics for evaluation are Precision, which is the average number of pixels correctly labeled, and Jaccard, which is the average intersection-over-union for foreground objects. Multiple Image Groups (MIG) [9] is a MSF-Coseg method that uses a multi-group image cosegmentation framework. This framework recognizes inter-image information, and transfers the information among the different groups in the datasets. They verified their method using Jaccard, and Precision. For our work, we will focus on previous works metrics, such as Precision and Jaccard, to homogenize the test. A summary of the cosegmentation methods that will be used in this paper is in Table 2.

**Table 2:** A condensed review of the three cosegmentation methods.

Model Name/Year	Cosegmentation Category	Supervised Level	Training Data	Components	Evaluation Metrics
*DeepCO^3^* [11] Year: 2019	DL-Coseg	Weakly-supervised	MS COCO [18], PASCAL VOC [17, 19], SOC [20]	VGG-16 [21], MatConvNet [22], ImageNet [23], ADAM [24]	CorLoc^r^, $mAP_{0.25}^{r}$, $mAP_{0.50}^{r}$
*Subdiscover* [10] Year: 2014	Cl-Coseg	Unsupervised	Internet [15]	Latent-SVM detector [25], NEIL [26]	Precision, Jaccard
*MIG* [9] Year:2016	MRF-Coseg	Unsupervised	iCoseg [14], Caltech-UCSD Birds 200 [27], Cat-Dog [28], Noise Image [15]	MRF [8], EM	IOU (a.k.a Jaccard) Precision

### Segmentation methods in plant phenotyping

In plant phenotyping, there are three common segmentation methods that are used when attempting to segment the foreground (plant) from the background. The first method is binary thresholding (also known as global thresholding) [29] where the image is converted to grayscale and the researcher determines the threshold number that will yield the most plant pixels. The second method, more advanced, is mean adaptive thresholding [29, 30] where the researcher looks at smaller portions of the object to determine the threshold number for each portion. However, with this method, the number of portions were determined beforehand with a trial and error approach that would yield the most plant pixels. If the portions were too small, it could lead to poor segmentation. Davies [29] found that this method only worked if the image had “nonuniform” lighting. The last method is Otsu thresholding [31] where the thresholding number is chosen to minimize the within-class variance. Adams [32] recently did work on plant phenotyping segmentation where he compared the three methods mentioned above with images taken in the same facility as *CosegPP*’s images. Based on Adams approach it was found that the three thresholding methodologies have no difference. Adams determined that since the lighting unclearly makes any part of the plant appear lighter, the performance of all three methods is similar. Therefore, this paper will use only Otsu’s thresholding in our comparison analysis since it is the common algorithm used by plant scientists due to its simplicity and automation (with knowledge that Otsu’s thresholding segmentation prevents certain component and holistic phenotypes).

## The *CosegPP* data repository

### Image acquisition

**Fig 1:**
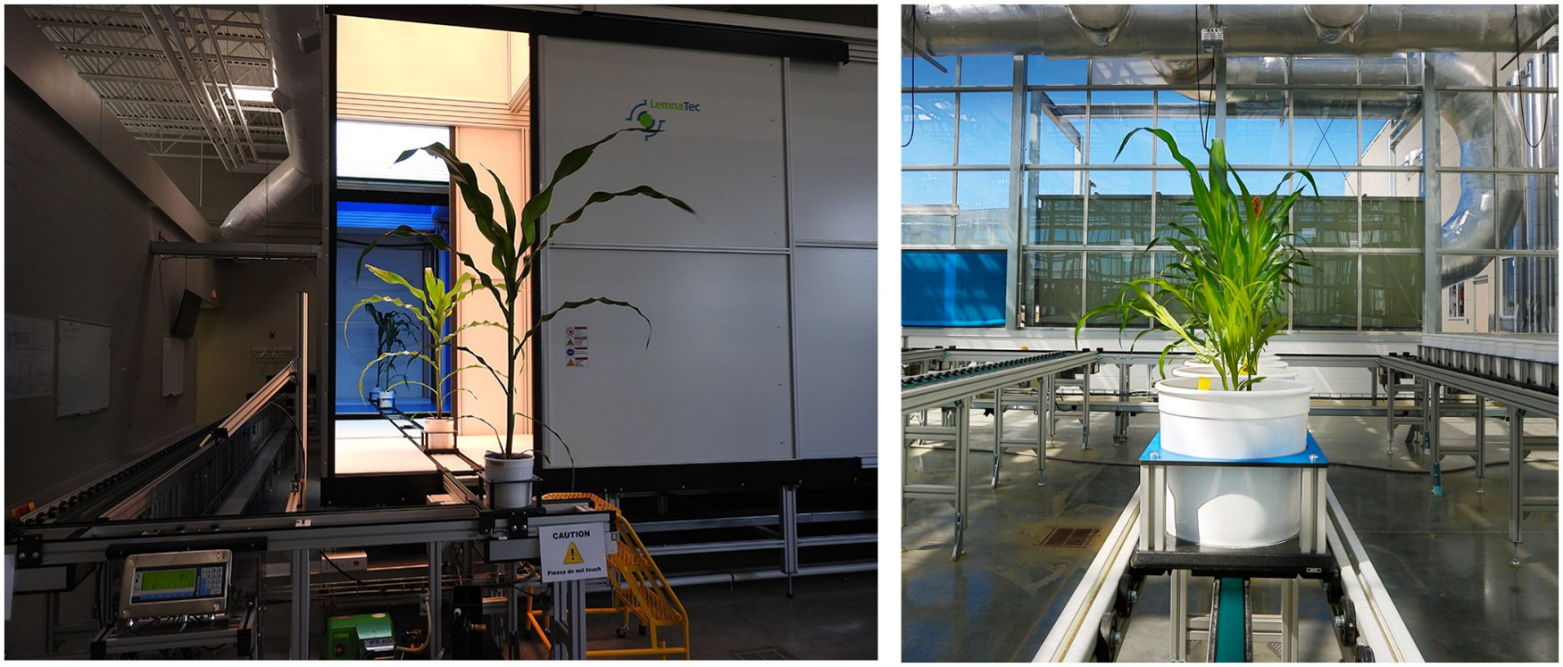
The proposed dataset, *CosegPP*, was collected using the LemnaTec Scanalyzer 3D plant phenotyping facility located at the University of Nebraska-Lincoln, USA. This facility is meant to create high throughput plant phenotyping datasets. The first image shows plants being imaged with a visible, infrared, fluorescent, and near infrared camera and exiting the LemnaTec chambers. The second image shows a row of plants on a conveyer belt in the greenhouse located next to the LemnaTec chamber.

*CosegPP* is derived from a larger dataset that contains an abundance of species (Sesame, Canna, Millet, Okra, Mo, etc.), modalities (infrared, near infrared, visible, and hyperspectral), more temporal points, and perspectives, and more experimental samples which were collected using the LemnaTec Scanalyzer at the University of Nebraska-Lincoln, USA (Fig 1). LemnaTec is a 3D high-throughput plant phenotyping system. The system transfers each plant through four imaging chambers in succession with attempts to be imaged daily. There is one camera type per chamber. Chamber one has the visible light (VIS) side-view (SV) and top-view (TV); chamber two has infrared (IR) SV and TV; chamber three has fluorescent (Fluo) SV and TV; and chamber four has near infrared (NIR) TV. Each imaging chamber has rotating lifters for up to 360-degree SV images.

*CosegPP* needed to be reorganized in the folder format that would be acceptable for the cosegmentation methods. The datasets were chosen based on:

1. having two physically different species for challenging segmentation. Buckwheat is a thin plant with a variety size of leaves and the Sunflower is a bushy plant that contains flowering;
2. having the most used induced environments in plant phenotyping such as a control and drought-induced;
3. having a temporal resolution that begins with the plants vegetative stage and ends with the plant fully matured;
4. having modalities (infrared, visible, near infrared) that are commonly used in plant phenotyping analysis; and
5. having multiple perspectives that are becoming widely acquired in plant phenotyping analysis due to its potential for three-dimensional analysis.

### Data organization

**Fig 2:**
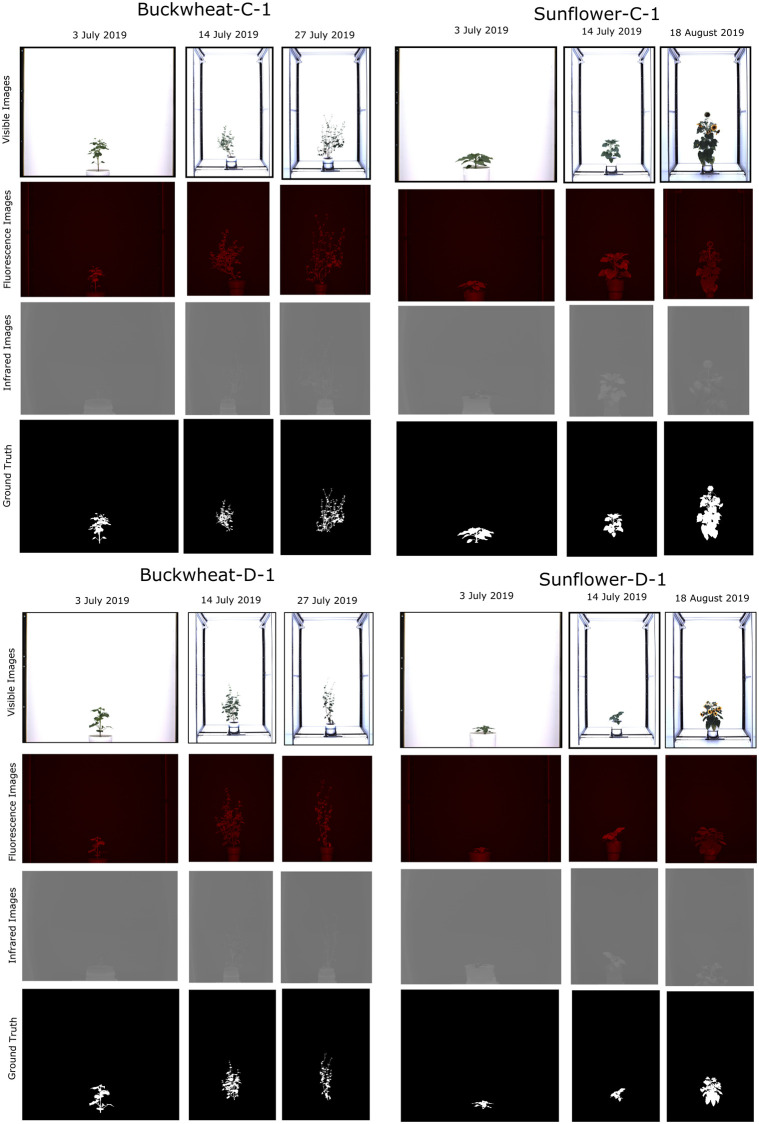
Preview of the proposed *CosegPP*. *CosegPP* is a data repository that contains four datasets: Buckwheat-C-1, Sunflower-C-1, Buckwheat-D-1, and Sunflower-D-1. The dataset names include the species name, C or D that represents Control or Drought induced, and a number representing the experimental repetition number. Each dataset includes visible, fluorescence, infrared, and ground truth images with 7 to 14 temporal images from 3 July to 18 August 2019. This preview shows only 3 temporal images in the 0-degree side view.

*CosegPP* (Fig 2) has Buckwheat-C-1, Buckwheat D-1, Sunflower-C-1, and Sunflower-D-1 as datasets. Dataset name starts with the name of the plant. C indicates control, D indicates drought, and 1 represents the plant ID number. Each dataset has 12 groups that are labeled with combinations of the three modalities (fluorescence, IR, Vis), perspectives (SV), and degree angles (0, 72, 144, 216) the photo was taken. Some example groups are: Fluo_SV_0, IR_SV_72, Vis_SV_144. Each group has a range of PNG images named after timestamps.

Ground truth images were obtained using Photoshop2020’s Action feature that pipelined the following actions: Quick Selection, Masking, Mode Conversion, Thresholding, and Inversion. After a binary mask was produced, two computer scientists checked each binary mask’s quality and added or removed pixels to produce a final binary mask. A binary mask was produced for each timestamp for each modality and perspective. Previous works have also used Photoshop in full or partial manual techniques when generating binary masks [12, 33–35].

## Benchmark experiment setup

### Benchmark protocols

We evaluated three existing cosegmentation datasets: iCoseg [14], MSRC [13], and Internet [15]. Between the three datasets, there are 20 groups totaling 156,688 images. For a fair analysis, we run the available code of the chosen cosegmentation methods with their default settings (including retraining DeepCO^3^ per dataset run). Modifications were made to the GPU arrays to handle *CosegPP*. No cropping was done on the images in *CosegPP* in order to challenge some of the cosegmentation methods on their claim of higher performance “with complex and diverse intra-class variations and background clutters” [11]. These experiments will evaluate three cosegmentation models: DeepCO^3^ [11], Subdiscover [10], and MIG [9] and one plant phenotyping segmentation model: Otsu’s thresholding [31], against *CosegPP* (ours), iCoseg [14], MSRC [13], and Internet [15].

### Evaluation metrics

For the evaluation, we will use Precision (P) (the average number of pixels correctly labeled) and Jaccard (J) (average intersection-over-union for the foreground objects) for segmentation accuracy since previous works in cosegmentation [6, 9, 36–38] has also used Precision and Jaccard. These metrics will be used to evaluate cosegmentation performance.

Let

$$\begin{array}{c} D=\{G_{1},\ldots ,G_{\text{k}},\ldots ,G_{\text{q}}\}\\ \begin{array}{cc} \text{where}\; & D\;\text{is}\;\text{the}\;\text{whole}\;\text{dataset}\\ & q\;\text{is}\;\text{the}\;\text{q}\text{th}\;\text{image}\;\text{group}\;G_{k}\\ & I_{i}^{k}\;\text{is}\;\text{the}\;\text{i}\text{th}\;\text{image}\;\text{in}\;\text{image}\;\text{group}\;G_{k} \end{array} \end{array}$$ (1)

Let

$$\begin{array}{c} G_{\text{k}}=\{I_{1}^{k},\ldots ,I_{i}^{k},\ldots ,I_{N_{k}}^{k}\}\\ \begin{array}{cc} \text{where}\; & N_{k}\;\text{is}\;\text{the}\;\text{number}\;\text{of}\;\text{images}\;\text{in}\;\text{the}\;G_{k}\\ & N_{D}\;\text{is}\;\text{the}\;\text{total}\;\text{number}\;\text{of}\;\text{images}\;\text{in}\;\text{the}\;\text{whole}\;\text{dataset}\;D \end{array} \end{array}$$ (2)

Each metric will have its own calculated *mean* score *ϑ* ∈ {*P*, *J*} for each dataset. The mean metric of each dataset will be defined as

$$\begin{array}{c} Q_{\vartheta }\left(D\right)=\frac{1}{N_{D}}\sum_{i=1}^{q}\sum_{k=1}^{N_{i}}\vartheta \left(I_{i}^{k}\right) \end{array}$$ (3)

Furthermore, we will also provide the *group mean* score defined as

$$\begin{array}{c} T_{\vartheta }\left(G_{i}\right)=\frac{1}{N_{i}}\sum_{k=1}^{N_{i}}\vartheta \left(I_{i}^{k}\right) \end{array}$$ (4)

## Quantitative comparisons

These analyses were computed at the Holland Computing Center (HCC) at the University of Nebraska-Lincoln. We were able to compute all cosegmentation and segmentation methods in the HCC using the Tesla V100 GPU nodes with Omni-Path Architecture using an average 190GB of RAM. HCC also has the capability to use CUDA, Docker, MATLAB, Matcaffe, and singularity, which are all needed to run the previous works’ code.

### Performance on the iCoseg dataset

iCoseg is a dataset that has a variety of group objects such as animals, landmarks, and sports. Table 3 shows the result’s Precision and Jaccard values per group for each cosegmentation and segmentation method.

**Table 3:** Results of three cosegmentation methods and one plant phenotyping segmentation method on the iCoseg dataset.

	DeepCO^3^		MIG		Subdiscover		Otsu Thresholding	
	P	J	P	J	P	J	P	J
*Christ*	0.75	0.58	**0.81**	**0.68**	**[]**	[]	0.48	0.32
*HotBalloons*	0.53	0.32	0.92	0.60	**0.96**	**0.68**	0.21	0.02
*Kendo*	0.55	0.35	**0.96**	**0.87**	0.85	0.56	0.05	0.02
*Kendo2*	0.69	0.46	**0.98**	**0.91**	0.90	0.61	0.02	0.00
*Liverpool*	0.56	0.25	0.84	**0.50**	**0.88**	0.14	0.43	0.07
*Monks*	0.64	0.43	**0.90**	**0.77**	[]	[]	0.53	0.26
*StatueofLiberty*	0.64	0.44	**0.94**	**0.74**	0.88	0.62	0.09	0.06
*TrackandField*	**0.72**	**0.31**	0.51	0.22	[]	[]	0.44	0.19
*Windmill*	0.64	0.16	0.78	**0.38**	**0.87**	0.25	0.57	0.21
*WomanSoccer*	0.68	0.35	0.90	**0.66**	**0.91**	0.54	0.33	0.08
*WomanSoccer2*	0.81	0.42	0.85	**0.51**	**0.87**	0.44	0.50	0.19
*baseball*	0.45	0.15	0.71	0.35	**0.96**	**0.62**	0.62	0.27
*bear2*	0.51	0.29	**0.77**	**0.53**	[]	[]	0.29	0.06
*brown_bear*	0.70	0.51	**0.90**	**0.71**	[]	[]	0.40	0.18
*cheetah*	0.66	0.48	**0.87**	**0.59**	[]	[]	0.57	0.34
*elephant*	0.50	0.24	0.57	0.32	**0.89**	**0.59**	0.36	0.03
*ferrari*	0.72	0.46	**0.89**	**0.64**	[]	[]	0.47	0.05
*goose*	0.72	0.47	0.84	0.58	0.81	0.38	**0.95**	**0.80**
*gymnastic1*	**0.90**	**0.58**	0.19	0.05	[]	[]	0.90	0.48
*gymnastic2*	**0.89**	**0.51**	0.38	0.15	[]	[]	0.85	0.37
*gymnastic3*	0.85	0.51	0.85	**0.61**	[]	[]	**0.90**	0.54
*helicopter*	0.43	0.20	0.98	0.79	**0.99**	**0.88**	0.18	0.04
*panda1*	0.63	0.47	**0.86**	**0.75**	0.78	0.48	0.48	0.25
*panda2*	0.59	0.48	**0.77**	**0.63**	0.61	0.32	0.50	0.33
*pyramid*	0.86	0.52	0.77	0.45	**0.95**	**0.80**	0.22	0.03
*skate*	0.81	0.60	**0.96**	**0.91**	[]	[]	0.15	0.04
*skate2*	0.35	0.10	0.77	0.48	**0.97**	**0.72**	0.04	0.01
*skate3*	0.39	0.11	0.51	0.11	**0.86**	**0.19**	0.26	0.02
*stonehenge*	0.87	0.73	**0.93**	**0.88**	0.77	0.50	0.43	0.26
*taj_mahal*	0.82	0.47	0.76	0.45	**0.84**	**0.49**	0.62	0.29
**All**	0.66	0.40	**0.79**	**0.56**	0.55	0.33	0.43	0.19

P is Precision, J is Jaccard, and [] is no data available. Bold text represents the highest score of both Precision and Jaccard per group. “All” is the average score on the whole dataset.

DeepCO^3^, at a glance, shows to not have acquired the highest scores for a majority of the groups. However, *TrackandField*, *gymnastics1*, and *gymnastics2* were the groups with the lowest number of images: 5, 6, and 4, respectively. This demonstrates the advantage of DeepCO^3^ has on groups with a low number of images as long as the object is similar despite its position/stance. All three groups had objects as people that wore the same uniform and were of same skin color. The only difference was the poses/perspective views.

MIG acquired the highest average Precision value and Jaccard for the iCoseg dataset at 0.7889 and 0.5606, respectively. MIG performs best when the object color is consistent while using SIFT features [39]. The groups with the highest Precision and Jaccard score (*Kendo2* and *skate*) all contain objects that are similar in size and color.

Subdiscover was not able to compute a segmentation mask for some of the groups (*Christ*, *Monks*, *bear2*, *brown_bear*, *cheetah*, *gymanstics1*, *gymanstics2*, *gymnastics3*, and *skate*). Despite that some of the group’s objects were not relatively in the same location of each image. Thus, having the segmentation masks include some noise, justifying the high precision and low Jaccard for all groups.

It is worth noting that the *goose* group achieved the highest Precision and Jaccard result from the Otsu Thresholding. An explanation of the *goose*’s group accuracy is that the object of the images (goose) is predominantly white. When thresholding, it is able to accurately segment the object due to the contrast between the predominately white goose and the blue water background.

iCoseg’s animal groups exhibit multiple features such as perspective and temporal. Having demonstrated that MIG performed well on 5 out of 7 animal groups (with the exception of the *elephant* and *goose* group) shows the potential of its capability to handle datasets that have at least multi-perspective and temporal features. Furthermore, the *goose* group is an excellent representation of *CosegPP*’s bimodal color images where Otsu Thresholding performed fairly well in achieving high segmentation accuracy. These advantages can be leveraged for our multi-feature *CosegPP*.

### Performance on the Internet dataset

The Internet dataset is focused on three objects: *Airplane*, *Car*, and *Horse*, demonstrating the difference between having 100 images of an object versus 4,300-6,300 images of an object. Table 4 shows the results of the Internet dataset when analyzed against the cosegmentation and segmentation algorithms.

**Table 4:** Results of three cosegmentation methods and one plant phenotyping segmentation method on the Internet dataset.

	DeepCO^3^		MIG		Subdiscover		Otsu Thresholding	
	P	J	P	J	P	J	P	J
*Airplane*	0.77	0.27	0.70	0.41	**0.90**	**0.61**	0.45	0.17
*Airplane100*	0.56	0.28	0.67	0.37	**0.90**	**0.50**	0.49	0.18
*Car*	0.73	0.49	0.76	0.59	**0.88**	**0.72**	0.45	0.21
*Car100*	0.77	0.62	0.75	0.58	**0.90**	**0.70**	0.45	0.20
*Horse*	0.74	0.28	0.70	0.42	**0.86**	**0.58**	0.45	0.13
*Horse100*	0.56	0.30	0.70	0.40	**0.89**	**0.48**	0.46	0.13
**All**	0.69	0.37	0.71	0.46	**0.89**	**0.60**	0.46	0.17

P is Precision and J is Jaccard. Bold text scores indicate the highest score of both Precision and Jaccard per group. “All” is the average score on the whole dataset.

DeepCO^3^ performed well in the *Car* and *Car100* group versus the others. It is worth noting that the Internet dataset had collections of other objects in a group. For example, the *Airplane* group had airplane, helicopter, and outliers as objects. The *Horse* group contained both fake and real horses. These objects not only are visually different, they are texturally different, as well.

MIG uses K nearest-neighbor search to calculate the foreground distance measurement. With the Internet dataset, the backgrounds are inconsistent which allows MIG to confuse parts of the background as the object. Although Internet contains similar objects, the background scenario is considered to be different in each image for MIG to segment properly.

The Subdiscover algorithm was verified using the Internet dataset. It is apparent in Table 4 where it shows the biases the Internet dataset has towards Subdiscover resulting in tight clusters (low Jaccard, high Precision). Subdiscover exploits dataset’s group objects being monochrome and in the same relative area in the image (center). It is worth noting that in the work of Subdiscover [9], the Internet dataset produced a Precision of.9042 (number converted to follow our paper’s format) in the *Car* group. Our results show 0.8809 Precision score for the *Car* group. The reason for the difference in the Precision score is because Subdiscover uses NEIL [26]. This object discovery and segmentation algorithm is constantly generating new segmentations using web data.

Otsu Thresholding performed the worst of all the algorithms due to the Internet dataset’s versatile background containing multiple colors, noise objects, and outlier images.

For each group in Internet, there are vast object variety images (different model/species types) and outlier images (starkly different from the object). Having groups with object variety images are similar to the multi-species and multi-environment feature where the object can be visually different towards the end of an experiment due to its induced environment. Subdiscover proves to be the superior method for these types of groups, and that can handle outlier images. In plant phenotyping, there are cases where some outlier images are captured such as an empty pot, a damaged plant, or inaccurate camera zoom.

### Performance on the MSRC dataset

MSRC’s groups mostly have 30 to 32 images except for the cat group at 24 images. Table 5 shows the Precision and Jaccard scores for the MSRC dataset against the cosegmentation and segmentation algorithms.

**Table 5:** Results of three cosegmentation methods and one plant phenotyping segmentation method on the MSRC dataset.

	DeepCO^3^		MIG		Subdiscover		Otsu Thresholding	
	P	J	P	J	P	J	P	J
*bike*	**0.68**	**0.45**	0.68	0.27	0.68	0.25	0.35	0.17
*bird*	0.49	0.22	**0.92**	**0.59**	[]	[]	0.51	0.23
*car*	0.65	0.52	0.69	0.38	**0.80**	**0.55**	0.37	0.19
*cat*	0.51	0.32	**0.84**	**0.50**	0.80	0.20	0.43	0.16
*chair*	0.57	0.34	0.79	**0.39**	**0.83**	0.37	0.48	0.24
*cow*	0.58	0.35	**0.93**	**0.74**	0.90	0.60	0.33	0.15
*dog*	0.49	0.29	**0.86**	**0.54**	[]	[]	0.43	0.19
*face*	0.65	0.48	0.80	0.57	**0.84**	**0.60**	0.51	0.29
*flower*	0.67	0.47	**0.82**	**0.63**	0.72	0.32	0.69	0.47
*house*	0.65	0.49	**0.83**	**0.63**	0.71	0.33	0.45	0.22
*plane*	0.61	0.30	0.84	0.51	**0.89**	**0.53**	0.59	0.19
*sheep*	0.57	0.36	**0.93**	**0.77**	0.90	0.65	0.67	0.33
*sign*	0.70	0.47	**0.86**	**0.63**	0.85	0.61	0.60	0.36
*tree*	0.74	0.58	0.66	0.44	**0.79**	**0.64**	0.31	0.18
**All**	0.61	0.40	**0.69**	**0.40**	**0.69**	**0.40**	0.48	0.24

P is Precision, J is Jaccard, and [] is no data available. Bold text represents the highest score of both Precision and Jaccard per group. “All” is the average score on the whole dataset.

DeepCO^3^ obtained the highest Precision and Jaccard score for only one group: *bike*. Although the Jaccard score was 0.4455, the *bike* group does contain a complex object with an array of different defining features.

MIG performed best in the *cow* and *sheep* group since both groups contain consistent backgrounds with similar sized objects.

In this dataset, Subdiscover was unable to identify clusters for the *bird* and *dog* class. Looking at the images within the two groups, it is understandable that the cosegmentation method struggled since both classes included a variety of species within the images. For example, the *bird* class has peacocks, swans, ducks, seagulls, pigeons, etc. All these birds have different physical features.

None of the groups had a high value for any of the metrics in the Otsu Thresholding algorithm. That indicates that MSRC’s images within the groups have multiple colors in the foreground and background.

MSRC has similar group objects as Internet and iCoseg where it contains object variety images, objects with multiple perspectives, and objects with temporal features (mostly for the animal groups). This dataset helps reiterate the claim that MIG and Subdiscover do well with multi-species, multi-perspective, multi-environment, and temporal features.

### Summary performance of iCoseg, internet, and MSRC

**Fig 3:**
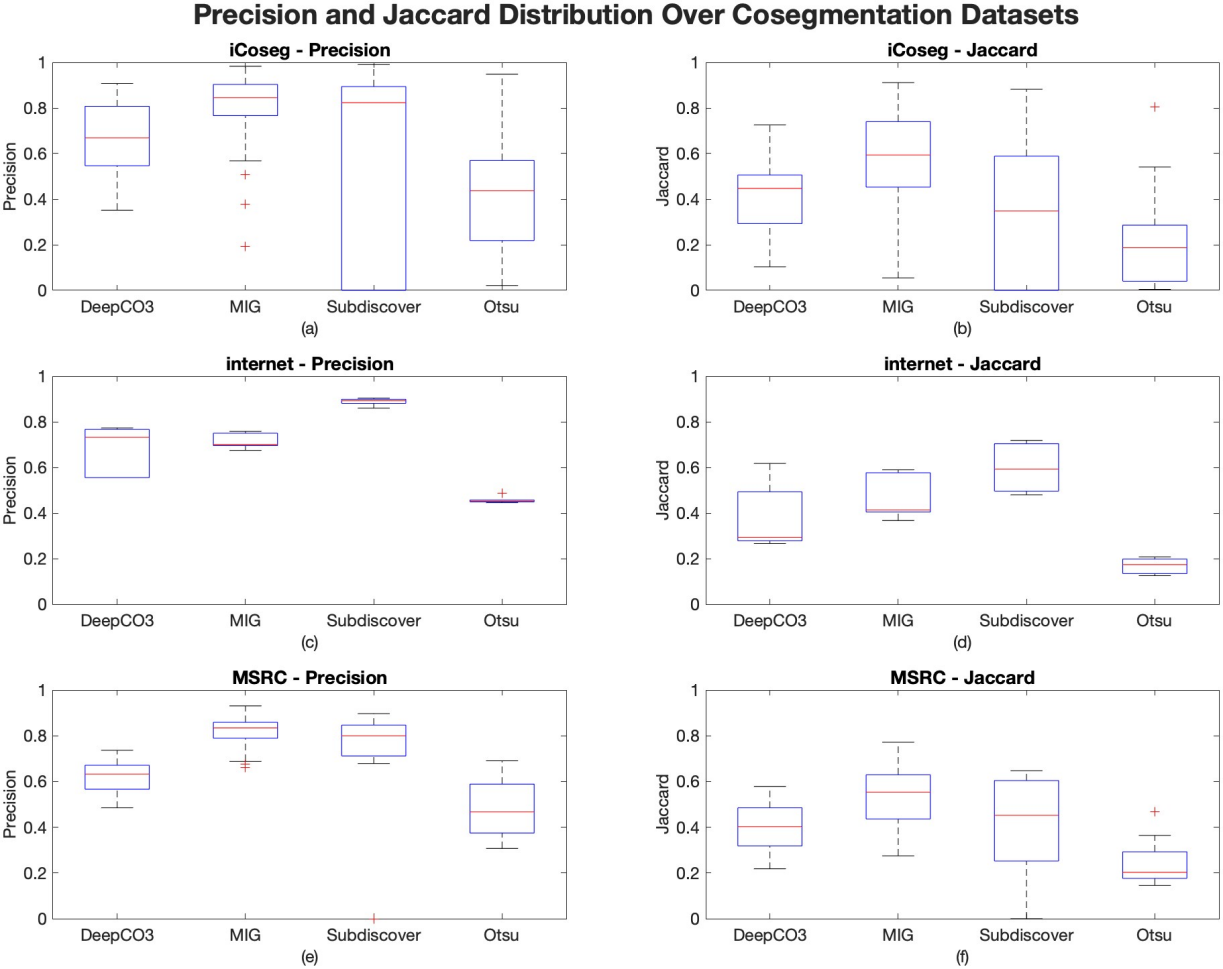
Precision and Jaccard distribution over all the cosegmentation datasets from the benchmark analysis. (a) and (b) are Precision and Jaccard distributions for iCoseg, respectively. (c) and (d) are the Precision and Jaccard distributions for Internet, respectively. (e) and (f) are the Precision and Jaccard distributions for MSRC, respectively.

Fig 3 displays a summary of all the Precision and Jaccard performance values in each cosegmentation dataset. These distributions reaffirm the claim that each dataset performed best in one or two of the tested methods without having a large range and the capability of detecting outliers.

### Performance on the *CosegPP* repository

Detailed results of our *CosegPP* analyses are shown in Table 6.

**Table 6:** Results of three cosegmentation methods and one segmentation method on *CosegPP*.

		DeepCO^3^		MIG		Subdiscover		Otsu Thresholding	
		P	J	P	J	P	J	P	J
Buckwheat-C-1	*Fluo_SV_0*	**0.98**	0.00	**0.98**	0.00	[]	[]	0.97	**0.38**
	*Fluo_SV_144*	**0.98**	0.00	**0.98**	0.0000	[]	[]	0.97	**0.38**
	*Fluo_SV_216*	**0.98**	0.00	**0.98**	0.00	[]	[]	0.97	**0.38**
	*Fluo_SV_72*	**0.98**	0.00	**0.98**	0.00	[]	[]	0.97	**0.36**
	*IR_SV_0*	0.98	0.00	**0.98**	0.00	[]	[]	0.91	**0.08**
	*IR_SV_144*	0.98	0.00	**0.98**	0.00	[]	[]	0.90	**0.05**
	*IR_SV_216*	0.97	0.00	**0.97**	0.00	[]	[]	0.89	**0.03**
	*IR_SV_72*	0.98	**0.07**	**0.98**	0.00	[]	[]	0.91	0.07
	*Vis_SV_0*	0.91	0.00	0.15	0.01	**0.99**	**0.30**	0.93	0.15
	*Vis_SV_144*	0.93	0.01	0.14	0.00	**0.99**	**0.28**	0.93	0.14
	*Vis_SV_216*	0.80	0.13	0.15	0.01	**0.99**	**0.22**	0.93	0.15
	*Vis_SV_72*	0.78	0.00	0.14	0.01	**0.99**	**0.56**	0.93	0.14
	**All**	**0.94**	0.02	0.70	0.00	0.33	0.11	0.94	**0.19**
Buckwheat-D-1	*Fluo_SV_0*	**0.98**	0.00	0.98	0.00	[]	[]	0.97	**0.38**
	*Fluo_SV_144*	0.97	0.00	0.97	0.00	[]	[]	**0.97**	**0.38**
	*Fluo_SV_216*	0.97	0.00	0.97	0.00	[]	[]	**0.97**	**0.38**
	*Fluo_SV_72*	0.97	0.00	0.97	0.00	[]	[]	**0.97**	**0.36**
	*IR_SV_0*	**0.96**	0.05	0.97	0.00	[]	[]	0.91	**0.08**
	*IR_SV_144*	0.96	0.01	**0.96**	0.00	[]	[]	0.90	**0.05**
	*IR_SV_216*	0.96	0.00	**0.96**	0.00	[]	[]	0.89	**0.03**
	*IR_SV_72*	0.96	0.06	**0.96**	0.00	[]	[]	0.91	**0.07**
	*Vis_SV_0*	0.76	0.00	0.15	0.01	**0.98**	**0.23**	0.93	0.15
	*Vis_SV_144*	0.59	0.01	0.15	0.01	**0.98**	**0.17**	0.93	0.14
	*Vis_SV_216*	0.80	0.00	0.15	0.01	**0.97**	**0.43**	0.93	0.15
	*Vis_SV_72*	0.59	0.01	0.15	0.01	**0.96**	0.12	0.93	**0.14**
	**All**	0.87	0.01	0.69	0.00	0.32	0.08	**0.94**	**0.19**
Sunflower-C-1	*Fluo_SV_0*	0.53	0.07	**0.97**	0.00	0.83	0.13	0.87	**0.30**
	*Fluo_SV_144*	0.60	0.07	**0.97**	0.00	0.86	0.10	0.87	**0.27**
	*Fluo_SV_216*	0.63	0.07	**0.97**	0.00	0.82	0.09	0.87	**0.28**
	*Fluo_SV_72*	0.86	0.13	**0.97**	0.00	0.88	0.08	0.86	**0.21**
	*IR_SV_0*	0.92	0.06	0.92	0.00	**0.93**	0.24	0.91	**0.34**
	*IR_SV_144*	0.93	0.04	0.93	0.00	**0.94**	0.21	0.91	**0.31**
	*IR_SV_216*	0.94	0.02	0.95	0.00	**0.95**	0.14	0.91	**0.29**
	*IR_SV_72*	0.94	0.04	0.94	0.00	**0.95**	0.19	0.92	**0.31**
	*Vis_SV_0*	0.70	0.17	0.26	0.04	**0.98**	**0.50**	0.93	0.33
	*Vis_SV_144*	0.73	0.12	0.26	0.04	**0.98**	**0.52**	0.92	0.31
	*Vis_SV_216*	0.78	0.13	0.26	0.04	**0.98**	**0.51**	0.92	0.31
	*Vis_SV_72*	0.75	0.11	0.25	0.03	**0.98**	**0.37**	0.92	0.26
	**All**	0.78	0.08	0.72	0.01	**0.92**	0.26	0.90	**0.29**
Sunflower-D-1	*Fluo_SV_0*	**0.99**	**0.19**	0.99	0.00	0.87	0.02	0.87	0.14
	*Fluo_SV_144*	0.91	0.12	**0.99**	0.00	0.84	0.01	0.87	**0.12**
	*Fluo_SV_216*	0.92	0.06	**0.99**	0.00	0.85	0.00	0.87	**0.13**
	*Fluo_SV_72*	0.99	0.07	**0.99**	0.00	0.82	0.00	0.87	**0.12**
	*IR_SV_0*	**0.97**	0.12	0.97	0.00	0.97	0.04	0.94	**0.28**
	*IR_SV_144*	0.98	0.01	**0.98**	0.00	0.98	0.09	0.93	**0.18**
	*IR_SV_216*	0.98	0.01	**0.98**	0.00	0.98	0.13	0.92	**0.17**
	*IR_SV_72*	0.98	0.01	**0.98**	0.00	0.98	0.12	0.93	**0.20**
	*Vis_SV_0*	0.71	0.05	0.21	0.02	**0.96**	0.12	0.93	**0.16**
	*Vis_SV_144*	0.73	0.04	0.21	0.01	**0.98**	**0.05**	0.93	0.14
	*Vis_SV_216*	0.74	0.01	0.21	0.02	**0.99**	**0.32**	0.93	0.15
	*Vis_SV_72*	0.80	0.07	0.21	0.01	**0.99**	**0.15**	0.93	0.13
	**All**	0.89	0.06	0.73	0.01	**0.93**	0.10	0.91	**0.16**

P is Precision, J is Jaccard, and [] is no data available; therefore, it will be treated as zero. Bold text represents the highest score of both Precision and Jaccard per group. “All” is the average score on the whole dataset.

DeepCO^3^ was unable to properly segment a majority of the groups in all four datasets since the Jaccard for all groups was nearly 0 except for *Fluo_SV_0* in the Sunflower-D-1 dataset and *Vis_SV_216* in the Buckwheat-C-1 dataset. Each Sunflower dataset had a total of 168 images (14 in each group) and each Buckwheat dataset had a total of 84 images (7 in each group). DeepCO^3^ claims to be able to segment where each group has 10 images. Therefore, *CosegPP* demonstrates that DeepCO^3^ has complications in properly segmenting the object despite the number of images per group.

Similarly, MIG was unable to segment all the fluorescence and infrared groups for all datasets. Although it was able to segment the visible group, it is near zero. MIG starts its computations by performing single image segmentation. If the single image segmentation results in no output or low Jaccard, the rest of the MIG algorithm fails when attempting to do single group and multiple group segmentation.

Buckwheat-D-1 is similar to Buckwheat-C-1 where Subdiscover was not able to segment the fluorescence and infrared modality. This could be due to the branches being too thin in Buckwheat. Subdiscover scored the highest in Precision and Jaccard for all the dataset’s visible group.

All the datasets had their best Jaccard score for the fluorescence and infrared group with Otsu Thresholding. Otsu Thresholding performed relatively well in these modalities since the grayscale intensities are relatively bimodal after converting the RGB image to grayscale. The plant images have dominantly two colors (green for the plant and white for the background). As the object of interest gets smaller compared to the background area, then the histogram will no longer exhibit bimodality [40].

In summary, Subdiscover continues to demonstrate accurate segmentation for a group of images that contain variety (plant youngling to plant maturity), but only for the visible modality. MIG performed similarly to Otsu thresholding in the Fluorescence and Infrared modality both demonstrating the ability to segment a group of images with multiple perspectives and temporal data.

An averaging was done across each feature for all datasets in *CosegPP* to determine which algorithms scored the highest for Precision and Jaccard as shown in Table 7. The final summary table is in Table 8.

**Table 7:** Averages of each feature in the *CosegPP* data repository.

		DeepCO^3^		MIG		Subdiscover		Otsu Thresholding	
		P	J	P	J	P	J	P	J
Modality	*Fluorescence*	0.89	0.05	**0.98**	0.00	0.42	0.03	0.92	**0.29**
	*Infrared*	0.96	0.03	**0.96**	0.00	0.48	0.07	0.91	**0.16**
	*Visible*	0.76	0.05	0.19	0.02	**0.98**	**0.30**	0.93	0.19
Perspective	*0*	0.87	0.06	0.71	0.01	0.63	0.13	**0.92**	**0.23**
	*144*	0.86	0.04	0.71	0.01	0.63	0.12	**0.92**	**0.21**
	*216*	0.87	0.04	0.71	0.01	0.63	0.15	**0.92**	**0.20**
	*72*	0.88	0.05	0.71	0.01	0.63	0.13	**0.92**	**0.20**
Species	*Buckwheat*	0.91	0.01	0.70	0.00	0.33	0.10	**0.94**	**0.19**
	*Sunflower*	0.83	0.07	0.72	0.01	**0.93**	0.17	0.91	**0.23**
Condition	*Control*	0.86	0.05	0.71	0.01	0.63	0.19	**0.92**	**0.24**
	*Drought*	0.88	0.04	0.71	0.00	0.63	0.08	**0.92**	**0.18**

P is Precision, J is Jaccard. Bold text represents the highest score of both Precision and Jaccard per group.

**Table 8:** Summary of the algorithms with the highest Precision and Jaccard for each feature in *CosegPP*.

		P	J
Modality	*Fluorescence*	MIG	Otsu Thresholding
	*Infrared*	MIG	Otsu Thresholding
	*Visible*	Subdiscover	Subdiscover
Perspective	*0*	Otsu Thresholding	Otsu Thresholding
	*144*	Otsu Thresholding	Otsu Thresholding
	*216*	Otsu Thresholding	Otsu Thresholding
	*72*	Otsu Thresholding	Otsu Thresholding
Species	*Buckwheat*	Otsu Thresholding	Otsu Thresholding
	*Sunflower*	Subdiscover	Otsu Thresholding
Condition	*Control*	Otsu Thresholding	Otsu Thresholding
	*Drought*	Otsu Thresholding	Otsu Thresholding

P is Precision, J is Jaccard.

### Summary performance of *CosegPP* repository

**Fig 4:**
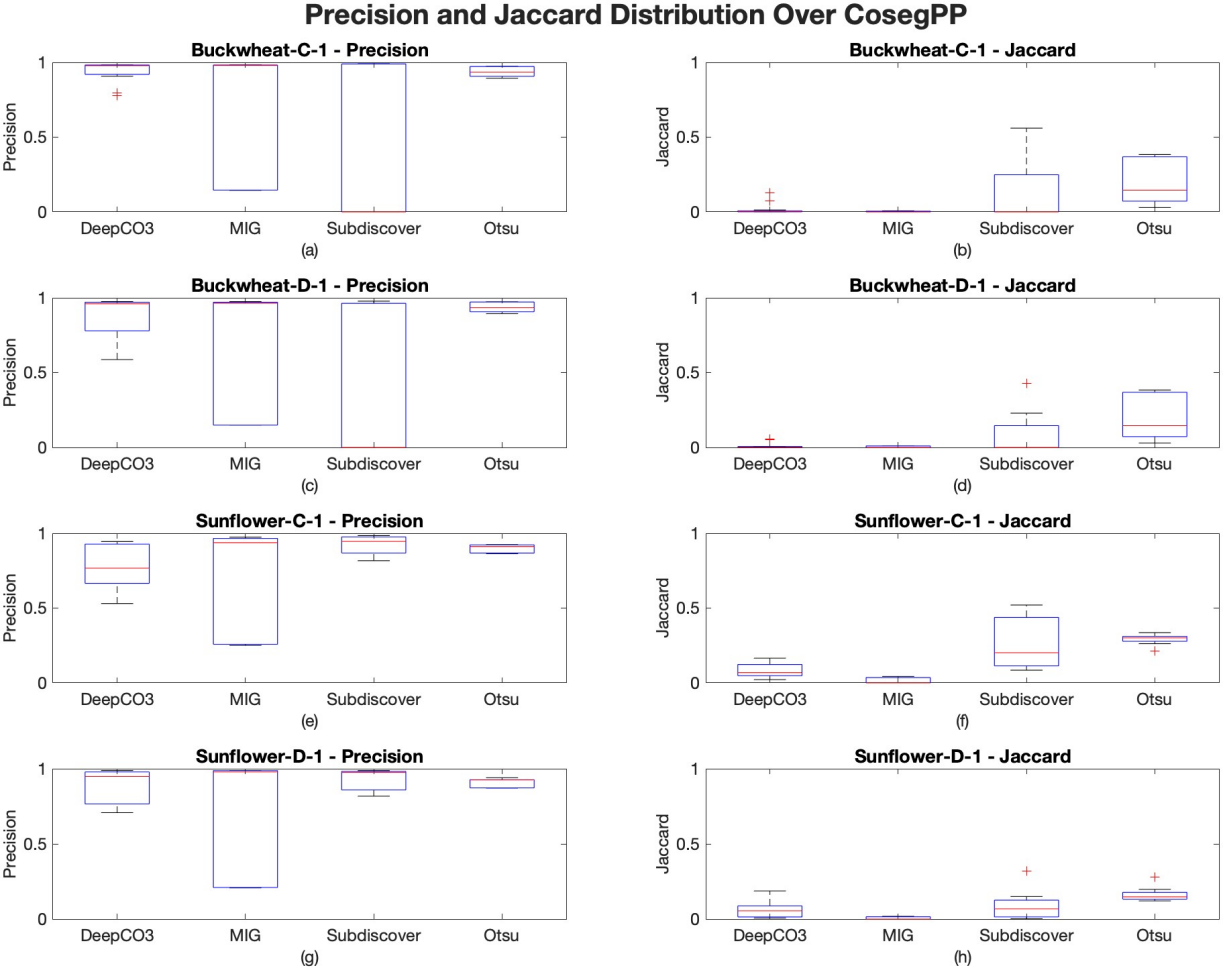
Precision and Jaccard distribution over *CosegPP* from the benchmark analysis. (a) and (b) are Precision and Jaccard distributions for Buckwheat-C-1, respectively. (c) and (d) are the Precision and Jaccard distributions for Buckwheat-D-1, respectively. (e) and (f) are the Precision and Jaccard distributions for Sunflower-C-1, respectively. (g) and (h) are the Precision and Jaccard distributions for Sunflower-D-1, respectively.

Fig 4 demonstrates that all cosegmentation methods, including the plant phenotyping segmentation method, had difficulties processing *CosegPP* due to its multiple features. Currently, there is no cosegmentation method that gives a promising performance for segmenting plant phenotyping multi-feature datasets. Even Otsu’s thresholding, despite it having the best performance, still has difficulties in overall segmentation resulting in less than 0.5 Jaccard.

By using a collection of methods for each feature in *CosegPP*, as shown in Table 8, the benefits can be leveraged to achieve higher segmentation accuracy.

## Qualitative comparisons

**Fig 5:**
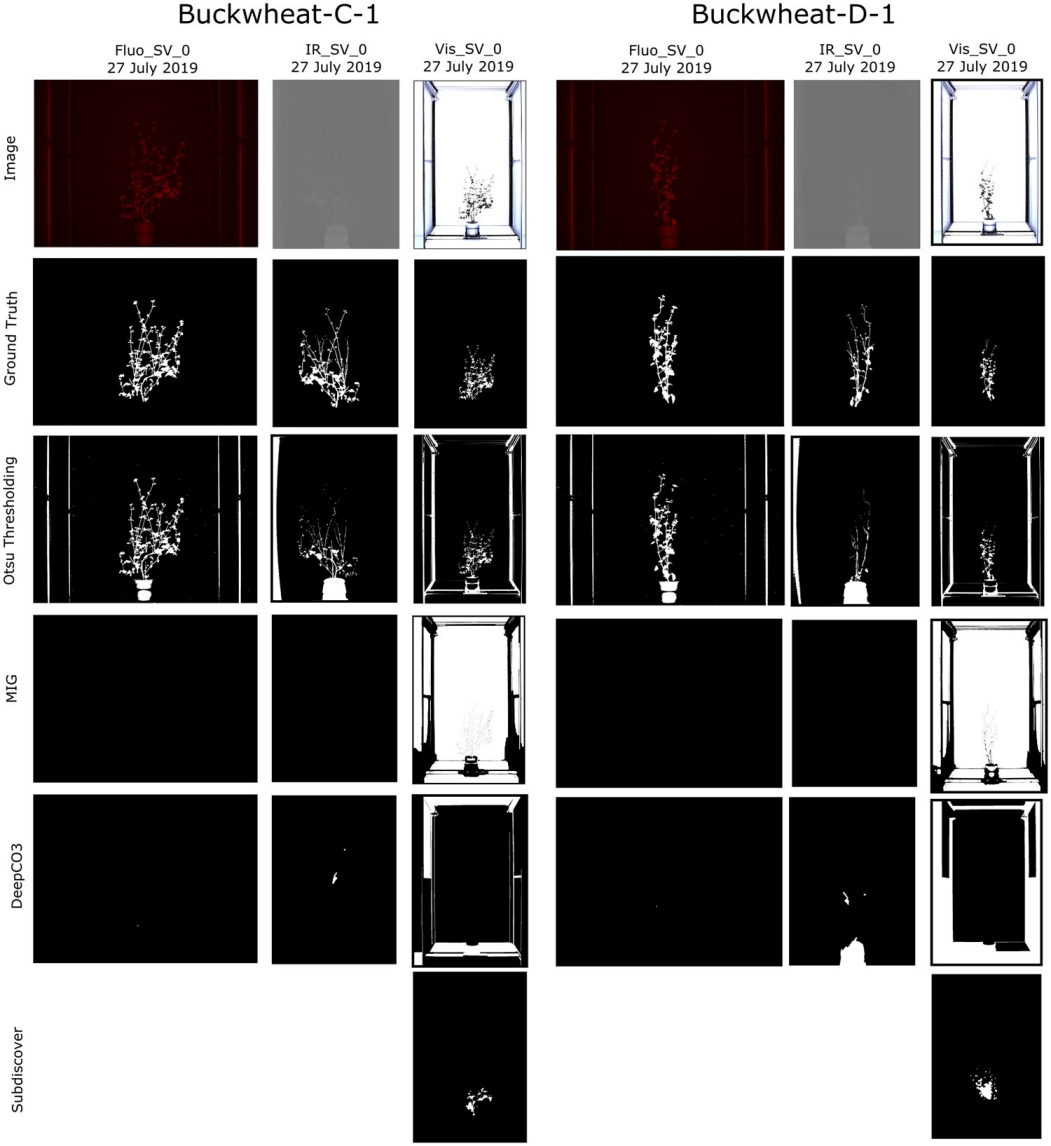
Qualitative performance examples of *CosegPP*’s Buckwheat with three cosegmentation methods (DeepCO^3^, MIG, Subdiscover) and one plant phenotyping segmentation methods (Otsu Thresholding). Each binary mask produced is the output of each cosegmentation methods with *CosegPP* where the white pixels are the pixels for the identified object(s). Subdiscover was unable to produce a binary mask for Buckwheat-C-1 and Buckwheat-D-1 in the fluorescence and infrared modality.

**Fig 6:**
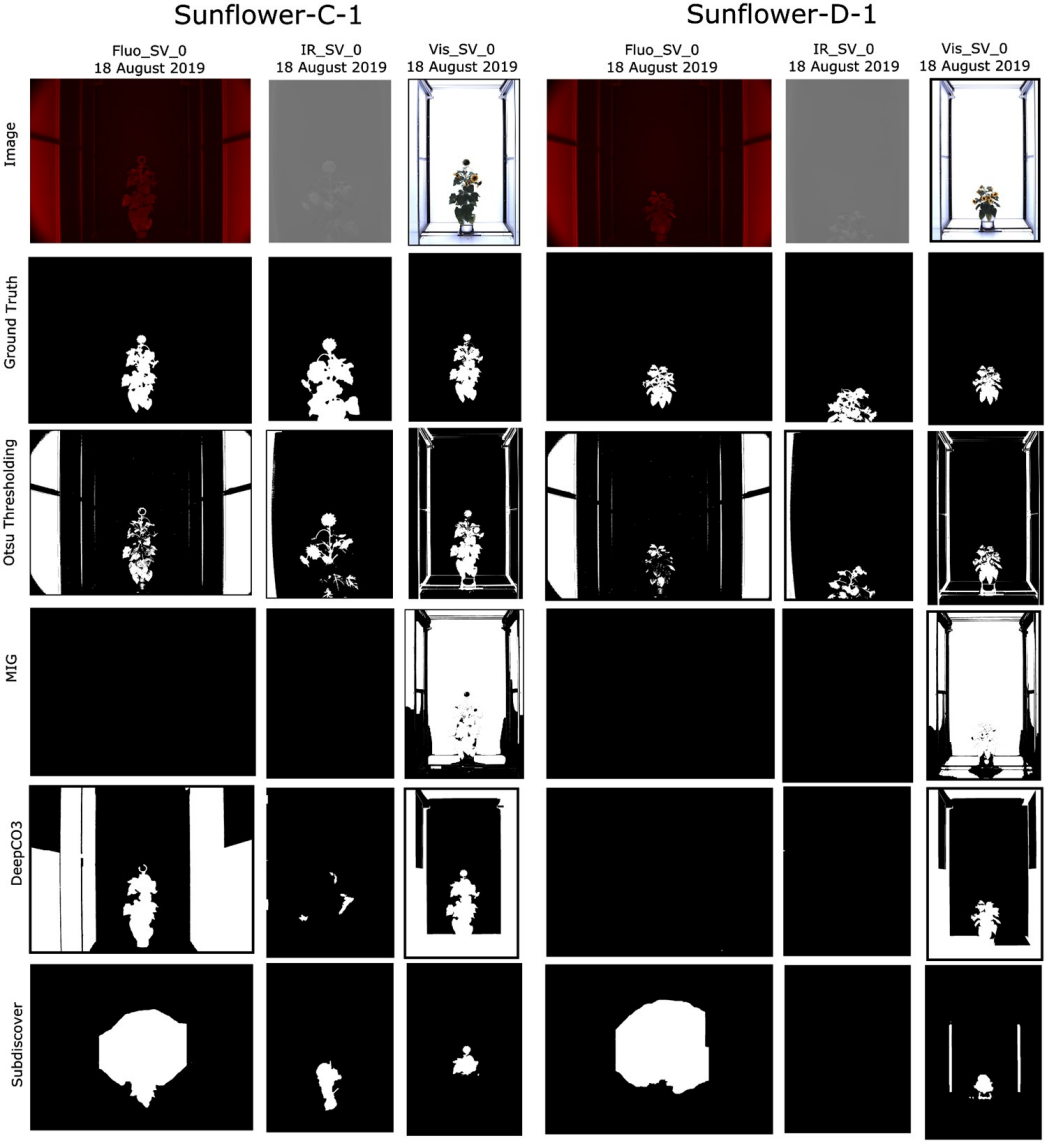
Qualitative performance examples of *CosegPP*’s Sunflower with three cosegmentation methods (DeepCO^3^, MIG, Subdiscover) and one plant phenotyping segmentation methods (Otsu Thresholding). Each binary mask produced is the output of each cosegmentation methods with *CosegPP* where the white pixels are the pixels for the identified object(s).

Figs 5 and 6 shows the visual results of *CosegPP* against MIG, DeepCO^3^, Subdiscover, and Otsu Thresholding. Notice how Subdiscover has empty slots in Buckwheat-C-1 and Buckwheat-D-1’s Fluo_SV_0 and IR_SV_0.

Looking at the images of Otsu Thresholding, the method was capable of segmenting both the Buckwheat and Sunflower plant relatively well. However, it also segmented parts of the LemnaTec chamber in which the tests were conducted. This is not an ideal segmentation for plant phenotyping since it will produce inaccurate phenotype results. Even in computer science, this is not an ideal segmentation since more than the targeted object was segmented.

MIG was only capable of segmenting the visible light images for both Buckwheat and Sunflower. Even though it segmented a majority of the LemnaTec chamber, it is worth noticing that the outline of each plant was ignored. MIG has the potential to generate an accurate object outline if complimented.

DeepCO^3^ was not able to segment Buckwheat from the images regardless of modality. It was only able to segment parts of the LemnaTec chambers in the visible light modality. Most likely due to the branch thinness and leaf thickness of the Buckwheat plant. Interestingly, DeepCO^3^ was capable of segmenting the Sunflower plant in the fluorescence and visible group in the control feature along with the visible modality in the drought feature.

Subdiscover was only able to segment the visible modality in both Buckwheat samples. Subdiscover seems to have segmented a visual “blob” of the overall location of the Sunflower plant and did a more accurate segmentation in the visible modality. Even though the segmentation was not accurate, it was able to determine the general location of the object via the blob which is a technique that can be leveraged to find neighboring plant pixels.

## Discussion

High-throughput phenotyping is a technique that has shown promising development to replace at least some traditional methods that are currently being used in plant phenotyping to access traits [5]. This is a field that must begin to transition from manual data acquisition and analyses to automatic, efficient hardware and software for resilient and sustainable farming. This study creates plant phenotyping datasets based on data obtained with a high-throughput imaging system that allows the analysis of multiple factors (multi-species, multi-perspective, multi-conditions, multi-modality, and temporal).

It is expected that the current cosegmentation datasets (iCoseg, Internet, and MSRC) had a bias in performance (Tables 3–5) for a single cosegmentation method. iCoseg performed the best with MIG, Internet with Subdiscover, and MSRC with MIG. This study demonstrates that each cosegmentation method leverages only 1-2 features (multi-perspective, and multi-species) restricting the type of datasets it can process.

This paper contributes *CosegPP* to further challenge cosegmentation by introducing multiple features to reduce biases. When using our dataset with the four methods, it is apparent that the cosegmentation methods performed equally or worse than the Otsu Thresholding for plant phenotyping (Tables 6–8). The quantitative and qualitative results shows that the cosegmentation methods have some computer science downfalls in not being able to 1) properly detect the full object (a.k.a. plant); and 2) ignore the cluttered background (a.k.a. LemnaTec chamber edges). It is possible that conducting some pre-processing on the images, such as cropping, can significantly improve the segmentation performance. However, having an algorithm smart enough to ignore the background will be more beneficial to the computer science and plant phenotyping field.

This paper also contributes a comparative study that suggests combining aspects of some or all the algorithms to improve segmentation accuracy with *CosegPP*. The ability for cosegmentation methods to accurately segment not only temporal images, but use a variety of modalities for inter-group information passing, along with different species, has the potential to introduce novel phenotypes are beneficial for plant scientists.

This study shows the benefit in the plant phenotyping community by demonstrating that image cosegmentation has the potential to increase phenotyping accuracy. More so, there are also benefits in the computing community by introducing a dataset that creates new challenges in a computer vision algorithm. Future work would be to evolve cosegmentation methodology to handle plant specific datasets to increase phenotyping accuracy to help address the problem of intensifying sustainable food production.

## Conclusion

This paper presents a complete group-level segmentation performance analysis using cosegmentation. These results identify a serious data bias, *i.e*., if each group of images contains similar visual appearances of the objects in current cosegmentation datasets. We created four new datasets within *CosegPP* that challenges the latest cosegmentation algorithms by having 1) a temporal component of plant growth; 2) different modalities for a variety of data type; and 3) introduction of varying colors and textures in the plant based on control or drought conditions. Our datasets combined total 330 images not including ground truth data. *CosegPP* is derived from a larger dataset containing a higher variety of species, temporal data, and an extra modality. Therefore, *CosegPP* has the potential to be expanded further to challenge the latest cosegmentation methods. Our datasets provide a leap in object physical characteristic diversity. Furthermore, this paper provided a comprehensive benchmark analysis that contains three of the latest cosegmentation methods and one segmentation method in plant phenotyping. These results provide a deeper analysis as to the issues and downfalls of the cosegmentation methods. Future work would be to create a new self-learning algorithm using multiple cosegmentation methods and coupling it with plant phenotyping ideologies to increase the segmentation accuracy in color, texture, and size changing objects.
